# Engaging Partners to Initiate Evaluation Efforts: Tactics Used and Lessons Learned From the Prevention Research Centers Program

**Published:** 2007-12-15

**Authors:** Demia Sundra Wright, Lynda A Anderson, Ross C Brownson, Margaret K Gwaltney, Jennifer Scherer, Alan W Cross, Robert M Goodman, Randy Schwartz, Tom Sims, Carol R White

**Affiliations:** Centers for Disease Control and Prevention; Centers for Disease Control and Prevention and Rollins School of Public Health, Emory University, Atlanta, Georgia; Prevention Research Center at Saint Louis University School of Public Health, St. Louis, Missouri; COSMOS Corporation, Bethesda, Maryland (now with Abt Associates Inc, Bethesda, Maryland); COSMOS Corporation, Bethesda, Maryland; University of North Carolina at Chapel Hill, Chapel Hill, North Carolina; Tulane University School of Public Health and Tropical Medicine, New Orleans, Louisiana (now with Indiana University School of Health, Physical Education, and Recreation, Bloomington, Indiana); American Cancer Society, New England Division, Framingham, Massachusetts; West Virginia Bureau for Public Health, Charleston, West Virginia; University of Kentucky, Lexington, Kentucky

## Abstract

**Background:**

The Centers for Disease Control and Prevention's (CDC's) Prevention Research Centers (PRC) Program underwent a 2-year evaluation planning project using a participatory process that allowed perspectives from the national community of PRC partners to be expressed and reflected in a national logic model.

**Context:**

The PRC Program recognized the challenge in developing a feasible, useable, and relevant evaluation process for a large, diverse program. To address the challenge, participatory and utilization-focused evaluation models were used.

**Methods:**

Four tactics guided the evaluation planning process: 1) assessing stakeholders' communication needs and existing communication mechanisms and infrastructure; 2) using existing mechanisms and establishing others as needed to inform, educate, and request feedback; 3) listening to and using feedback received; and 4) obtaining adequate resources and building flexibility into the project plan to support multifaceted mechanisms for data collection.

**Consequences:**

Participatory methods resulted in buy-in from stakeholders and the development of a national logic model. Benefits included CDC's use of the logic model for program planning and development of a national evaluation protocol and increased expectations among PRC partners for involvement. Challenges included the time, effort, and investment of program resources required for the participatory approach and the identification of whom to engage and when to engage them for feedback on project decisions.

**Interpretation:**

By using a participatory and utilization-focused model, program partners positively influenced how CDC developed an evaluation plan. The tactics we used can guide the involvement of program stakeholders and help with decisions on appropriate methods and approaches for engaging partners.

## Background

Established by a Congressional mandate in 1984, the Prevention Research Centers (PRC) Program comprises 33 university-based research centers that address the most important chronic disease and health promotion issues facing public health today. With funding from the Centers for Disease Control and Prevention (CDC), the PRCs conduct prevention research and training as well as disseminate and translate research into practice and policy (1,2). The PRC Program represents a national community of researchers and others with a vested interest in prevention research, including national public health organizations, universities with programs in public health and preventive medicine, state and local health departments, and community members. Starting with just three PRCs in 1986, the PRC Program produces practical, high-quality intervention research, training programs, and dissemination approaches that are grounded in the realities of communities across the United States (3).

In 2001, the PRC Program began Project DEFINE (Developing an Evaluation Framework: Insuring National Excellence), a 2-year evaluation planning project. Guided by the six-step framework (hereafter referred to as the CDC framework) outlined in *Framework for Program Evaluation in Public Health* (4), the PRC Program implemented the first two steps of the CDC framework for Project DEFINE: 1) engaging stakeholders and 2) describing the program through the development of a program logic model. The PRC Program staff at CDC engaged stakeholders to develop a logic model and final evaluation plan. The process allowed a variety of perspectives from across the national community of PRCs to be expressed and reflected. This case study presents the tactics used in, and lessons learned from, using a participatory approach for Project DEFINE.

## Context

Initially, the task seemed enormous. The CDC framework (4), however, provided a six-step strategy for evaluation planning that centers on four basic standards for program evaluation — utility, feasibility, propriety, and accuracy. These steps and standards laid the groundwork for a solid approach that engaged many interested partners — primarily the directors, researchers, staff, partners, and communities of the PRCs; staff from national and state partner organizations; and members of CDC leadership.

CDC staff recognized that a participatory approach could benefit the evaluation planning process and the PRC Program overall. Previous evaluations of CDC-funded and other programs show that engaging stakeholders and using participatory methods result in increased use of findings, increased relevance to stakeholders' needs, evaluation capacity building at the grantee level, decreased anxiety about the evaluation process, and support for future evaluation activities (5-8). In addition, the CDC framework and other sources state that focusing on an intended and meaningful use for the evaluation findings (i.e., taking a utilization-focused approach) leads to more relevant results and a sense of project ownership (4,7,8). For the PRC Program, the utilization-focused approach was fundamental because the PRC Program logic model would not only be used for program planning at CDC but would also provide the basis for logic models developed by individual PRCs for their own evaluations.

CDC staff in the PRC Program implemented the evaluation planning project through a contract that stipulated, on the basis of recommendations in the evaluation literature (4-8), that the process should be participatory and focused on use of a logic model by the PRCs and the PRC Program overall. The literature that informed the contract supported CDC's intention to 1) reduce the anxiety and skepticism that many PRCs felt about developing both a national evaluation and a single national program description for a large and diverse program; 2) build on the program knowledge and sense of program ownership among PRC directors, staff, and partners; 3) create buy-in among PRC partners to facilitate evaluation activities that would rely on their support and participation; 4) reflect the increased importance of partnerships and participatory research methods in the PRC Program; 5) produce more feasible evaluation designs and appropriate methods; and 6) build evaluation capacity within the PRCs.

## Methods

**Box:** Tactics Used to Guide Partner Engagement in the Prevention Research Centers (PRC) Program’s Project DEFINE (Developing an Evaluation Framework: Insuring National Excellence)

Tactics
1. Assess stakeholders' communication needs and existing communication mechanisms and infrastructure.
2. Use existing mechanisms, and establish others as needed to inform, educate, and request feedback.
3. Listen to and use the feedback received.
4. Obtain adequate resources, and build flexibility into the project plan to support multifaceted mechanisms for data collection.

The evaluation planning process formally began when the contract was awarded in 2001. The core evaluation work group comprised evaluation contractors and PRC Program staff. One of the first activities of the work group was to form an advisory committee, named the Collaborative Evaluation Design Team (CEDT). The core evaluation work group and CEDT worked closely throughout the project and learned several lessons as a result of the collaborative project activities. Realizing that these lessons could be helpful to other programs and responding to the support of reflective practice in the evaluation field (9), we (the core evaluation work group and the CEDT) reflected on the project and reviewed project documents. We then refined and came to consensus on four tactics that guided the planning process and led to the project's success (Box). The first and second tactics were explicit within the contract's scope of work, and we discuss how they were implemented and refined to engage stakeholders. The third and fourth tactics resulted from our observations on feedback from stakeholders and our retrospective assessment of the project. These four tactics respond to the recommendations in the CDC framework, the program evaluation standards for increasing the utility and support of a project, and the multiple perspectives of the partners in the PRC Program.

### Tactic 1: Assess stakeholders' communication needs and existing communication mechanisms and infrastructure

When initiating Project DEFINE, CDC communicated with PRC partners through the existing standing committees: an overall steering committee and five topic-specific committees. These committees, whose members are PRC representatives and provide input and guidance to CDC's PRC Program staff, were key to communicating with and seeking input on the evaluation design from PRC directors and other PRC leaders. Involving these committees had two benefits. First, because the PRCs elected the committee members, a committee stamp of approval on evaluation activities offered a form of peer endorsement. Second, one of the committees, the National Community Committee (NCC), included leaders from the PRCs' partnering communities, thereby ensuring the incorporation of community perspectives into the deliberations of the core evaluation work group as well as relaying important information on Project DEFINE to other community leaders involved with each PRC.

Because no PRC committee was devoted to an evaluation planning process, the CEDT was created to help guide the project and create additional avenues for stakeholder input. Consistent with the CDC framework (4), the CEDT included an array of perspectives and expertise from across the PRC Program. The PRCs nominated CEDT members, and the committee ultimately included representatives of the PRC directors, PRC staff, state health departments, community members, national partner organizations, and an evaluation expert experienced in conducting community-based research. The CDC project officer for Project DEFINE (L.A.A.) and a PRC director (R.C.B.) co-led the CEDT. Discussions between the core evaluation work group and the CEDT during monthly conference calls and semiannual in-person meetings were recorded in meeting minutes and summary documents, as were all written communications and feedback from participants.

The CEDT helped to ensure project relevance, feasibility, and utility and to facilitate input from stakeholders. The CEDT's involvement in decision making and project oversight enabled stakeholders to trust that the project would represent their perspectives and not just those of the core evaluation work group. Finally, the CEDT and core evaluation work group reflected on the lessons learned and made adjustments as the project unfolded.

### Tactic 2: Use existing mechanisms, and establish others as needed to inform, educate, and request feedback

The contract's scope of work outlined a series of participatory methods that were used during Project DEFINE. These methods led to the development of a program description in the form of a logic model that reflected input from all PRC Program partners. Concept mapping was the primary method used to develop the basic constructs of the logic model (10). Other methods used provided a deeper understanding of the context, program realities, and viewpoints of the PRCs, which are necessary to develop a logic model that accurately describes the program (11). In particular, two in-person methods were useful in eliciting partners’ perspectives on the PRC Program overall and the evaluation planning project specifically: 1) visiting six PRCs to understand contextual factors and 2) holding three regional meetings for obtaining feedback and encouraging open dialogue between partner groups. A written document that served as a structured feedback tool was then sent to four main groups (the PRCs, the NCC, state partners, and CDC’s PRC Program staff); the feedback tool allowed for comments on the final logic model and narrative. The core evaluation work group also consistently used other methods to inform various partner groups, provide opportunities for discussion, and obtain feedback on activities and products before broader release. These methods included presenting updates at the semiannual meetings of PRC directors and the meetings of other partner groups, participating in CEDT monthly conference calls, conducting semiannual in-person meetings, and discussing issues with members of the standing committees during their regularly scheduled calls or retreats.

Through these activities, stakeholders shared diverse perspectives, values, and priorities. The approach encouraged partners to work through challenges, such as the differences among stakeholders in program understanding, project expectations, and power differentials that are common in community–academic partnerships (12,13).

### Tactic 3: Listen to and use the feedback received

Because a transparent decision-making process, information sharing, and feedback for project changes are essential in partnerships and participatory projects (4,8,12,13), we paid careful attention to listening and responding to feedback from partners, to following through on recommendations, and to actively communicating the resulting changes. For example, the CEDT initially recommended developing two logic models for the PRC Program, one highlighting the national perspective and national-level outcomes and the second delineating the community perspective and community-level outcomes. After reviewing the two draft models at the regional meetings, representatives from all of the PRCs recommended that the models be combined. The core evaluation work group, with the CEDT's guidance, then developed a single logic model for the program that incorporated the ideas of community members, PRCs, and other partners and distributed the draft for review using a structured feedback tool. The example illustrates how the core evaluation work group was committed to responding to feedback in order to increase trust and buy-in. Seeing their ideas implemented confirmed for the partners that the project was genuinely aimed at creating products that were relevant and useful to all partner groups. Thus, partners experienced benefits of the participatory process and continued to participate in project activities.

### Tactic 4: Obtain adequate resources, and build flexibility into the project plan to support multifaceted mechanisms for data collection

A participatory project often has a more fluid process than a nonparticipatory project, and therefore funding organizations, researchers, or evaluators must be able to adjust project plans as needed (12,13). On the basis of partners' participation and input, Project DEFINE's direction changed several times, each time becoming more relevant and useful. For example, partners asked for another opportunity to review the logic model before it was finalized, particularly because the two logic models were being combined into one, and this step was added to the planning process. Development of a structured feedback tool for soliciting comments to be used in finalizing the national logic model was added to the contract.

Flexibility in the use of project resources was also an important factor in Project DEFINE. When the core evaluation work group modified plans, resources were also reallocated across tasks. CDC supported the project year by year. CDC's up-front commitment of both staff and funding ensured that the project was participatory, even though this approach to evaluation planning has higher costs and takes more time to complete (12,13). Foremost, however, the core evaluation work group recognized the importance of having dedicated project leaders and a committed group of partners who believed in the PRC Program and willingly gave their time and energy to the project.

## Consequences

The four tactics described allowed us to continually listen and provide feedback to our national community of researchers and others with a vested interest in prevention research. The processes also led to lessons learned, which we describe below.

### Benefits

Using participatory methods and having a utilization-focused approach for Project DEFINE resulted in several benefits. First, CDC's PRC Program office increased its abilities to strategically manage the program. The PRC Program's national logic model, a tangible product of the participatory process and available on the PRC Program Web site (3), was used to improve the 5-year cooperative agreement program announcement (14), protocols for CDC site visits to PRCs, and templates for grantee work plans and progress reports. These new materials not only reflect the perspectives of the PRCs but also assess partner engagement in all PRC activities. The data from Project DEFINE continue to be used in the development of a national evaluation protocol. The increased evaluation activities respond to CDC's accountability needs, addressing the long-term investment in the PRC Program by establishing mechanisms to understand how PRCs operate, their uniqueness and breadth, and the impact of the PRC Program overall.

Second, individual PRCs have increased their evaluation activities over the years. During the project, the involvement of PRC partners increased as they understood that CDC would use the logic model for planning and that their input could influence future program decisions. Since then, each PRC has been required to develop its own logic model and a related evaluation plan for the 5-year cooperative agreement application. Several PRCs have used these logic models for strategic planning and evaluation or have created logic models for specific research projects.

Third, academic, community, and state partners of the PRCs now expect to be engaged in PRC planning and research activities. The focus on community-based research has intensified during the 20-year history of the PRC program and is now explicit in the national program's requirements. Community partners have stated that they appreciate having their role more formally defined.

Fourth, unexpected benefits beyond the PRC program have also resulted, including adaptation of the PRC logic model by CDC's National Academic Centers of Excellence (ACE) on Youth Violence Prevention (15). The constructs and narrative description for the ACE logic model reflect input from ACE program partners and are consistent with the youth violence prevention research policies and Congressional language.

### Challenges

A primary challenge of the participatory process was the time and effort required for Project DEFINE. The work described here extended over 2 years. This time was necessary to engage the network of diverse program partners and address the complex issues involved in developing the logic model. A second challenge was the investment in program resources. The type of planning required for Project DEFINE can be costly, although methods can be tailored for various budgets and data requirements. A third challenge was identifying the critical project decision points for each step and the people to engage in feedback and decision-making processes. For example, when should program partners be consulted? When should CDC bring draft documents to the CEDT for comment? The best way to answer these questions was to communicate openly with members of the CEDT, asking for their perspective on the methods to use and the decisions in which they should be involved.  

## Interpretation

Having the infrastructure and processes in place to ensure routine and repeated communication with, and engagement of, stakeholders throughout the project was extremely valuable. By using a participatory model and staying attentive to the project's practical use, we were motivated to use a variety of methods to involve all partner groups throughout the process, all of which influenced the development of the national logic model. Consistent communication and commitment to bringing in diverse viewpoints from the PRCs and their partnering communities led to stakeholder support of, and involvement in, Project DEFINE. Ongoing feedback also served as a periodic touchstone to ensure that the project remained pertinent and responsive to the needs of all partners. The project established the groundwork that the PRC Program needed to prepare for national evaluation and created momentum to continually engage partners in these activities.

The CDC framework and its program evaluation standards offer a structure for planning a public health evaluation project and principles to follow as the project progresses (4). Participatory mechanisms and methods for obtaining feedback or sharing updates need to be tailored for individual projects and programs. The experiences from Project DEFINE offer examples for other programs that need to cohesively and effectively engage diverse partners and stakeholders. We hope that our reflections on this evaluation planning project help guide others engaging in large-scale public health program evaluations and assist those working to involve a broader representation of program stakeholders, whether for building an evaluation framework, for developing a logic model, or for other purposes. For the national community of researchers and partners involved in the PRC Program, Project DEFINE propelled the program forward in documenting how PRCs conduct prevention research and in assessing whether the program is having the intended impact on public health research, policy, and practice.
